# A Young Adult With Persistent Headache: A Case of Central Neurocytoma

**DOI:** 10.7759/cureus.77470

**Published:** 2025-01-15

**Authors:** Mohammed A Johar, Amal R Dhaif, Alaa K Mahdi, Shaimaa H Banaji, Anas Ahmed

**Affiliations:** 1 General Practice, Salmaniya Medical Complex, Manama, BHR; 2 College of Dentistry, Ajman University, Ajman, ARE; 3 College of Medicine, King Saud University, Riyadh, SAU; 4 College of Medicine, King Abdulaziz University, Jeddah, SAU; 5 College of Medicine, Jazan University, Jazan, SAU

**Keywords:** central neurocytoma, gross total resection, histopathology, intraventricular tumor, obstructive hydrocephalus, synaptophysin, young adult

## Abstract

Central neurocytomas are rare, typically benign neuronal tumors that primarily affect young adults and are most commonly located within the lateral ventricles. This report presents the case of a 23-year-old male who presented with a two-month history of progressive headache, nausea, vomiting, and cognitive decline. Neurological examination revealed papilledema, indicative of increased intracranial pressure. Magnetic resonance imaging of the brain demonstrated a well-defined, partially calcified intraventricular lesion with associated obstructive hydrocephalus. Histopathological analysis following a stereotactic biopsy confirmed the diagnosis of central neurocytoma, with immunohistochemistry showing positivity for synaptophysin and neuronal nuclear antigen. The patient underwent a successful gross total resection of the tumor, and postoperative magnetic resonance imaging confirmed complete removal with no residual disease. The patient’s recovery was uneventful, and he remains asymptomatic at six months post-surgery with no evidence of recurrence. This case underscores the importance of early recognition, accurate diagnosis, and timely surgical intervention in the management of central neurocytomas. Additionally, it highlights the favorable prognosis associated with gross total resection, although long-term surveillance remains essential to detect any recurrence.

## Introduction

Central neurocytoma is a rare, typically benign intraventricular tumor arising from neuronal progenitor cells. It accounts for approximately 0.1-0.5% of all central nervous system tumors and is predominantly observed in young adults between 20 and 40 years of age [1,2]. These tumors are most commonly located in the lateral ventricles near the foramen of Monro, often resulting in obstructive hydrocephalus due to ventricular outflow obstruction. The clinical presentation varies but typically includes symptoms of increased intracranial pressure, such as headache, nausea, vomiting, and visual disturbances. Cognitive and behavioral changes may also occur in some cases [3,4].

Histologically, central neurocytomas are characterized by small, round, uniform cells with neuronal differentiation. Immunohistochemical markers such as synaptophysin and neuronal nuclear antigen are crucial for diagnosis. Imaging studies, particularly magnetic resonance imaging (MRI), often reveal a well-defined, heterogeneous, partially calcified intraventricular mass with cystic and solid components [2-5].

Despite their generally favorable prognosis, central neurocytomas may recur, especially in cases of incomplete resection. The standard treatment is gross total surgical resection, often followed by adjuvant radiotherapy in cases of subtotal excision or higher proliferative activity. Long-term follow-up is essential to monitor for recurrence and ensure optimal outcomes [5,6].

## Case presentation

A 23-year-old male presented to the emergency department with complaints of persistent headache, nausea, and intermittent episodes of vomiting over the preceding two months. The headache was described as dull, predominantly in the frontal region, and exacerbated by physical activity or changes in position. There was no history of trauma, fever, seizures, visual disturbances, or limb weakness. The patient reported a progressive decline in concentration and occasional forgetfulness. His past medical history was unremarkable, and there was no significant family history of neurological disorders or malignancies. He was a non-smoker, did not consume alcohol, and was not on any regular medications.

On physical examination, the patient appeared alert and oriented but exhibited mild discomfort due to the headache. His vital signs were stable, with a blood pressure of 118/76 mmHg, heart rate of 72 bpm, and temperature of 36.7°C. Neurological examination revealed no focal deficits. Cranial nerve testing was normal, with intact visual fields and normal pupillary reflexes. Motor strength and deep tendon reflexes were symmetric, and sensory examination showed no abnormalities. Fundoscopic examination revealed bilateral papilledema, raising suspicion of increased intracranial pressure. Other systemic examinations were unremarkable.

Initial laboratory investigations were performed to rule out systemic causes of the patient’s symptoms and to establish baseline values for perioperative management. The results, along with the reference ranges, are summarized in Table 1. These results revealed no abnormalities in complete blood count, renal function, or electrolytes. Inflammatory markers such as C-reactive protein and erythrocyte sedimentation rate were within normal limits, effectively ruling out an infectious or systemic inflammatory process. The coagulation profile was also normal, ensuring the patient was an appropriate candidate for surgical intervention.

**Table 1:** Laboratory results and reference ranges

Test	Result	Reference Range
Hemoglobin	14.2 g/dL	13.5–17.5 g/dL
White blood cell count	6,200/µL	4,000–11,000/µL
Platelet count	245,000/µL	150,000–450,000/µL
Sodium	140 mmol/L	135–145 mmol/L
Potassium	4.3 mmol/L	3.5–5.0 mmol/L
Creatinine	0.85 mg/dL	0.74–1.35 mg/dL
Blood urea nitrogen	14 mg/dL	7–20 mg/dL
C-reactive protein	<1.0 mg/L	<5 mg/L
Erythrocyte sedimentation rate	8 mm/hr	<20 mm/hr
Prothrombin time	12.5 sec	11.0–13.5 sec
International normalized ratio	1.0	0.8–1.1
Glucose (fasting)	90 mg/dL	70–100 mg/dL

**Figure 1:**
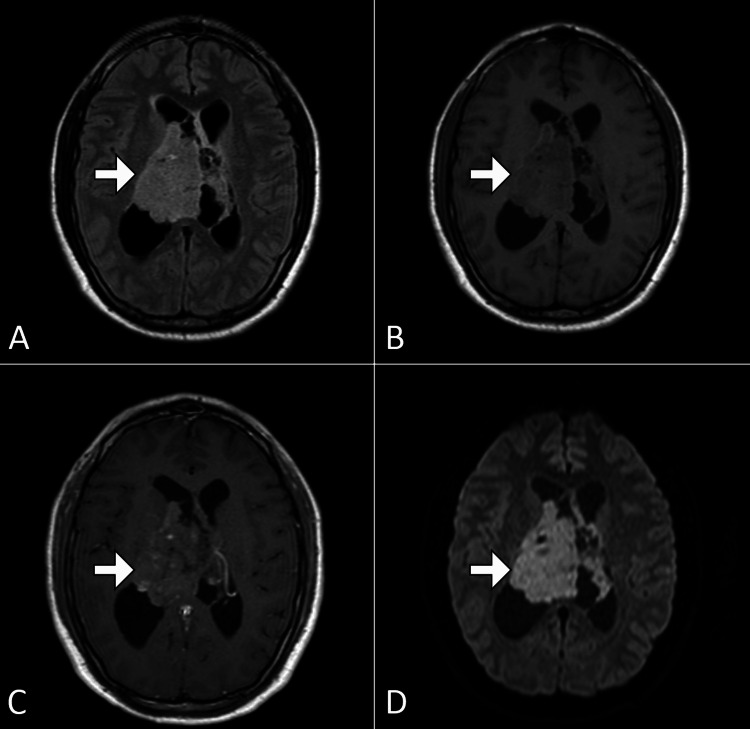
Axial MRI images of the brain Selected axial MRI images of the brain: FLAIR (A), T1-weighted (B), post-contrast T1-weighted (C), and DWI (D). The images reveal an ill-defined lesion (arrow) within the right lateral ventricle, centered on the septum pellucidum, causing obstructive hydrocephalus. MRI, magnetic resonance imaging; FLAIR, fluid-attenuated inversion recovery; DWI, diffusion-weighted imaging

**Figure 2:**
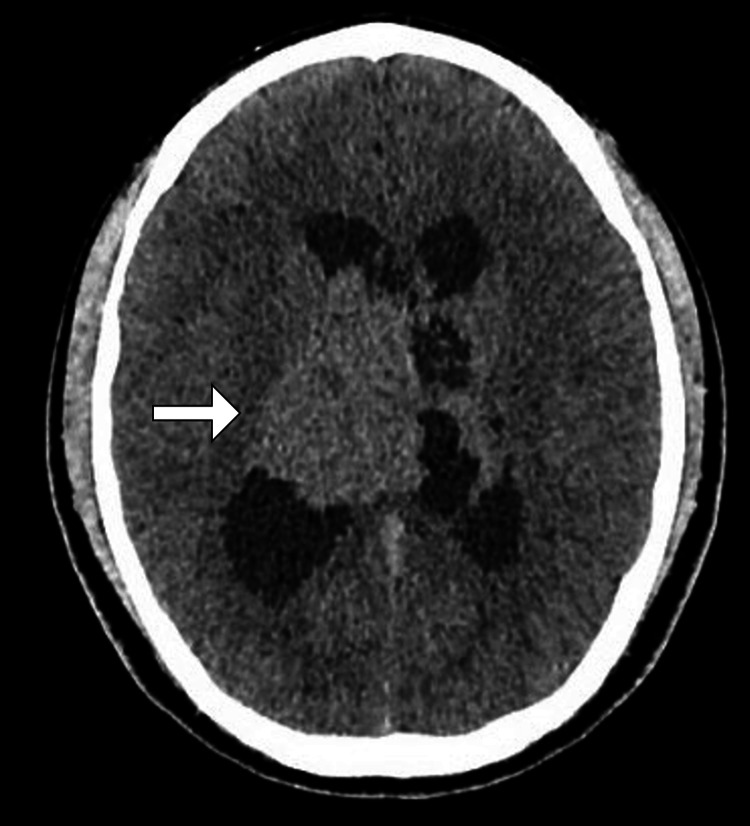
CT image of the brain Axial CT image of the brain demonstrating a slightly hyperdense lesion (arrow) within the right lateral ventricle with associated hydrocephalus. CT, computed tomography

MRI of the brain with contrast revealed a well-defined intraventricular lesion within the body of the left lateral ventricle. The lesion measured approximately 3.5 cm × 2.8 cm and exhibited heterogeneous enhancement with cystic components and areas of calcification. There was associated mass effect with compression of the foramen of Monro, leading to obstructive hydrocephalus. Diffusion-weighted imaging showed no restricted diffusion, and perfusion studies were suggestive of moderate vascularity within the lesion. No evidence of adjacent parenchymal invasion or extracranial extension was noted (Figure 1). Computed tomography demonstrated the lesion as having high attenuation (Figure 2).

Based on the imaging findings, the differential diagnosis included central neurocytoma, subependymoma, ependymoma, and intraventricular meningioma. The location within the lateral ventricle with a non-invasive pattern was suggestive of central neurocytoma as the primary consideration. To confirm the diagnosis, a stereotactic biopsy was planned.

The patient underwent endoscopic biopsy under general anesthesia. Histopathological examination of the tissue sample revealed small, round, uniform tumor cells arranged in a sheet-like pattern with focal pseudorosette formation. Immunohistochemical staining was positive for synaptophysin, neuronal nuclear antigen, and glial fibrillary acidic protein, consistent with the diagnosis of central neurocytoma. The Ki-67 labeling index was approximately 2%, indicating low proliferative activity.

Following the diagnosis, the patient was scheduled for surgical resection of the tumor. A craniotomy was performed, and gross total resection (GTR) was achieved with intraoperative neuro-navigation guidance. The patient’s recovery was uneventful, and he was discharged on the seventh postoperative day with instructions for routine follow-up.

At the one-month follow-up visit, the patient reported significant improvement in symptoms, with complete resolution of headache and nausea. Neurological examination remained normal, and repeat imaging showed no evidence of residual or recurrent tumor. The patient was referred to radiation oncology for discussion of adjuvant therapy, given the risk of recurrence, although it was deferred due to the low proliferative index and GTR.

The patient continues to be followed up regularly with periodic clinical evaluation. At six months post-surgery, he remains asymptomatic. His quality of life has improved significantly, and he has resumed his academic pursuits without any functional limitations.

## Discussion

Central neurocytoma is a rare, low-grade neuronal tumor that predominantly affects young adults, representing a small fraction of primary intracranial neoplasms [1-3]. This case highlights the typical clinical, radiological, and histopathological features of the tumor, along with the challenges and strategies involved in its management. The discussion aims to provide a comprehensive analysis of this condition in light of existing literature, underscoring the significance of timely diagnosis, multidisciplinary management, and long-term surveillance.

The patient in this case presented with symptoms of increased intracranial pressure, including headache, nausea, and papilledema, which are consistent with obstructive hydrocephalus - a hallmark clinical manifestation of central neurocytomas due to their intraventricular location. The predominance of these tumors in the lateral ventricles near the foramen of Monro makes them prone to causing ventricular outflow obstruction. Early neuroimaging plays a pivotal role in identifying such lesions, with MRI being the modality of choice [3,5]. The imaging characteristics of central neurocytomas, such as a well-defined, heterogeneous mass with cystic changes, calcifications, and moderate enhancement, are crucial for differential diagnosis [4-7]. In our case, these imaging findings guided the initial suspicion of central neurocytoma, distinguishing it from other intraventricular tumors such as ependymomas, subependymomas, and meningiomas.

Histopathological confirmation remains the gold standard for diagnosis. The small, uniform cells with neuronal differentiation observed in our patient’s tumor, coupled with immunohistochemical positivity for synaptophysin and NeuN, are consistent with the defining features of central neurocytoma [2,6]. The low Ki-67 index (<5%) in this case correlated with the tumor’s low proliferative potential and favorable prognosis. These findings align with existing data, where the Ki-67 index is often used as a prognostic marker to stratify the risk of recurrence and guide adjuvant therapy decisions.

Surgical resection remains the cornerstone of treatment for central neurocytomas, with GTR being the goal to achieve optimal outcomes [4,5]. In our case, GTR was successfully achieved, and postoperative imaging confirmed the absence of residual tumor. Studies have demonstrated that GTR significantly reduces the risk of recurrence and prolongs progression-free survival compared to subtotal resection [5-7]. However, the deep-seated location and proximity to critical neurovascular structures often limit the extent of resection, necessitating adjunctive therapies.

Adjuvant radiotherapy is recommended in cases of subtotal resection or tumors with a high proliferative index. While our patient did not receive radiotherapy due to the completeness of resection and low Ki-67 index, regular follow-up remains imperative to detect any potential recurrence [2-6]. Recent advances in conformal radiotherapy techniques, such as intensity-modulated radiotherapy and proton therapy, offer targeted treatment with minimal collateral damage, making them viable options for high-risk patients.

The prognosis for central neurocytoma is generally favorable, with five-year survival rates exceeding 90% following GTR. However, recurrence rates of 10-30% have been reported, emphasizing the need for long-term surveillance [2-5]. In our patient, the absence of symptoms and recurrence at six months post-surgery is encouraging yet highlights the importance of ongoing monitoring with clinical assessments and imaging.

This case underscores the importance of a multidisciplinary approach involving neurosurgery, neuropathology, radiology, and oncology for the optimal management of central neurocytomas. It also emphasizes the need for individualized treatment strategies based on tumor characteristics and resection status. Future research should focus on molecular profiling of central neurocytomas to identify potential biomarkers for prognostication and therapeutic targets. Additionally, prospective studies comparing surgical and adjuvant treatment modalities would further elucidate the optimal management strategies for this rare entity.

## Conclusions

Central neurocytoma is a rare intraventricular tumor that predominantly affects young adults and poses diagnostic and therapeutic challenges. Early recognition of its clinical and radiological features is crucial for timely intervention, as delayed diagnosis can lead to significant complications due to obstructive hydrocephalus. GTR remains the cornerstone of treatment, offering the best chance for long-term disease-free survival. Histopathological confirmation and immunohistochemical analysis are essential for accurate diagnosis and prognostication. While the overall prognosis is favorable, the potential for recurrence, particularly in cases of incomplete resection or higher proliferative activity, necessitates vigilant long-term follow-up. This case highlights the importance of a multidisciplinary approach and personalized care in achieving optimal outcomes for patients with central neurocytoma.

## References

[1] Chen CL, Shen CC, Wang J, Lu CH, Lee HT (2008). Central neurocytoma: a clinical, radiological and pathological study of nine cases. Clin Neurol Neurosurg.

[2] Dutta SW, Kaleem TA, Muller DA, Peterson J, Harrell AC, Quinones-Hinojosa A, Trifiletti DM (2018). Central neurocytoma: clinical characteristics, patterns of care, and survival. J Clin Neurosci.

[3] Lee SJ, Bui TT, Chen CH (2016). Central neurocytoma: a review of clinical management and histopathologic features. Brain Tumor Res Treat.

[4] Yang I, Ung N, Chung LK, Nagasawa DT, Thill K, Park J, Tenn S (2015). Clinical manifestations of central neurocytoma. Neurosurg Clin N Am.

[5] Patel DM, Schmidt RF, Liu JK (2013). Update on the diagnosis, pathogenesis, and treatment strategies for central neurocytoma. J Clin Neurosci.

[6] Imber BS, Braunstein SE, Wu FY (2016). Clinical outcome and prognostic factors for central neurocytoma: twenty year institutional experience. J Neurooncol.

[7] Choudhari KA, Kaliaperumal C, Jain A, Sarkar C, Soo MY, Rades D, Singh J (2009). Central neurocytoma: a multi-disciplinary review. Br J Neurosurg.

